# Wound Dressing and Postoperative Complications Following Breast Reduction: A Retrospective Study of 250 Patients

**DOI:** 10.1093/asjof/ojag131

**Published:** 2026-06-26

**Authors:** Steven L Zeng, Thomas Ren, Ricardo R Colon, Nina E Ringelman, Salman Choudhry, Gedge Rosson

## Abstract

**Background:**

Postoperative wound dressings are routine but understudied in reduction mammaplasty. Although an ideal dressing should promote healing, limit infection, and be well-tolerated, practice varies widely without consensus.

**Objectives:**

This study compares the impact of dressings on postoperative outcomes following reduction mammaplasty.

**Methods:**

A single-institution, retrospective review was performed of reduction mammaplasty patients. Data included demographics, operative details, dressing type, and complications. Dressings included Dermabond (DER), Prineo (PRI), Histoacryl (HIS), Sylke (SYL), Steri-Strips (STE), and combinations (COM). Contralateral breasts in oncoplastic or free-flap cases were excluded.

**Results:**

A total of 250 patients (439 breasts) were included. At baseline, cohorts differed in BMI, American Society of Anesthesiologist class, diabetes, operative time, and resection weight (all *P* < .05), largely because of PRI, which contained higher-risk patients. Analyses were repeated, excluding PRI: differences resolved. On univariate analysis, PRI had the highest complication rates. Excluding PRI, HIS had higher skin reaction rates than SYL (*P* = .003) and COM (*P* = .049), and higher dehiscence than SYL (*P* = .043). On multivariate analysis, SYL predicted lower skin reaction (odds ratio [OR] 0.21, *P* = .003) and dehiscence (OR 0.47, *P* = .02). PRI predicted higher reoperation (OR 6.4, *P* = .01) and poor scarring (OR 34.8, *P* < .001), although limited by baseline confounding.

**Conclusions:**

Dressing selection may affect wound outcomes after reduction mammaplasty. SYL tape was associated with lower rates of skin reaction and dehiscence across both analyses, although potentially limited by residual confounding. PRI's association with reoperation and poor scarring is notable but difficult to disentangle from patient selection bias. Combination dressings showed lower skin reaction rates but were heterogeneous, making interpretation challenging. Prospective data are needed to confirm these findings.

**Level of Evidence: 3 (Therapeutic):**

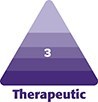

Reduction mammaplasty is commonly performed to address both the functional and aesthetic consequences of macromastia. Its prevalence has increased in recent years because of the substantial improvements in quality of life reported by patients, making it the seventh most performed procedure by plastic surgeons worldwide.^1^ Despite its high success rate, postoperative wound complications, such as dehiscence, delayed healing, and poor scarring, persist as frequent concerns.^2^

Postoperative dressing remains a critical yet sometimes underappreciated component of postoperative care following reduction mammaplasty. An ideal dressing should provide a protective barrier against bacterial contamination, maintains appropriate moisture balance, help distribute tension across the incision, and remain comfortable for the patient.^3^

Currently, there exists an abundance of postoperative dressing options in the surgeon's armamentarium. These include an assortment of surgical glues and adhesive bandages. Cyanoacrylates, such as Dermabond (DER; Ethicon, Somerville, NJ) and Histoacryl (HIS; B. Braun, Melsungen, Germany) have been found to decrease intraoperative closure times and lower operative cost when compared with subcuticular closures.^4,5^ However, multiple studies have reported incidences of contact dermatitis after cyanoacrylate use in breast procedures.^6-9^ Additionally, DER use has been associated with increased rates of wound dehiscence, hypertrophic scarring, and fat necrosis.^10^ DER Prineo (PRI; Ethicon), a 2-octyl cyanoacrylate skin adhesive applied over a polyester mesh for skin closure, has shown to lower infection rates and decrease operative time.^11^ However, the potential remains for similar local allergic reactions as seen with DER.^12^ Other traditional options include adhesive skin closure strips, such as Steri-Strips (STE; 3M, White City, OR), permit faster closure compared with subcuticular sutures alone, and provide comparable cosmetic outcomes to DER at a lower cost.^13,14^

Recent advances in postoperative dressings include silk-fibroin dressings, Sylke (SYL; (SYLKE, La Jolla, CA) and incisional negative pressure wound therapy. Purified silk fibroin is a biomaterial that is nonimmunogenic, highly biocompatible, and noncytotoxic to human skin tissue.^15,16^ Previous reviews have found it to have significantly lower rates of wound dehiscence and contact dermatitis when compared with STE and PRI, respectively.^17,18^ Incisional negative pressure wound therapy has also been a promising advancement for managing surgical incisions on the breast, touting lower rates of surgical-site infections, wound dehiscence, and wound necrosis compared with conventional dressings.^19^ In current practice, there remains no proven “best” option for wound dressings for reduction mammaplasty. Thus, the purpose of this study is to evaluate the effectiveness and postoperative morbidity of the most commonly used postoperative dressings at our institution, following reduction mammaplasty.

## METHODS

An IRB exempt retrospective chart review was conducted on all patients who underwent reduction mammaplasty at the Johns Hopkins Hospital from July 2023 to July 2025. Inclusion criteria were defined as patients aged 18 years or older who underwent bilateral or unilateral reduction mammaplasty. For patients undergoing oncoplastic reduction or free-flap reconstruction, the contralateral, nonreduction breast was excluded. Lastly, study participants received no funding, industry support, or have any conflicts of interest.

A comprehensive review of operative logs and progress reports was performed to extract patient demographics (eg, BMI, smoking status, allergies, and medical comorbidities), operative details (eg, weight of resection, drain usage, and previous skin glue exposure), wound dressings, and complications and reoperation rates. Cohorts were separated by postoperative wound dressing: DER, PRI, HIS, SYL, STE, and a combination of dressings (COM). Dressings were selected based on the clinical judgment and preference of the surgeon. A total of 6 surgeons were included in this cohort. Our primary outcome was defined as any major or minor postoperative complication. Major complications included reoperation, and minor complications included skin reaction (localized, erythema, blistering, and rash), hematoma/seroma, documented wound dehiscence, infection (purulence, cellulitis, and abscess), skin necrosis, need for antibiotics, and poor scarring (defined as hypertrophic scarring, keloid formation, hyperpigmentation, need for scar revision, or documented patient dissatisfaction with scar appearance).

### Statistical Analysis

Bivariate analyses were performed using standard analysis of variance for continuous variables, with Tukey's honestly significant difference test for post hoc pairwise comparisons. Pearson's χ^2^ test was used for categorical variables. Multivariate logistic regression was conducted to evaluate the association between patient demographics, operative details, and wound dressing type with each postoperative complication, using an enter-all approach informed by univariate results. Model performance was assessed using McFadden's pseudo-*R*^2^ and the Hosmer–Lemeshow goodness-of-fit test. All statistical analyses were performed using RStudio version 3.3.0 (R Foundation for Statistical Computing, Vienna, Austria), with significance defined as *P* < .05.

## RESULTS

A total of 250 patients, including 439 breasts, underwent reduction mammaplasty during the study period. The mean age was 40.2 ± 16.1 years, and the mean BMI was 32.0 ± 5.6 kg/m^2^. Current and former smoking patients were present in 6.6% and 10.7% of cases, respectively, and 7.7% of patients had diabetes mellitus. The mean resection weight was 609.3 ± 420.6 g on the left and 622.7 ± 423.9 g on the right, with an average operative time of 171.4 ± 65.1 min. Drains were placed in 29.4% of patients. History of previous skin glue use was seen in 25.6% of patients, and there were no reported adhesive allergies. The mean follow-up duration was 97 ± 124 days. Patient demographics and operative variables are represented in Table 1.

**Table 1.** Patient Demographics and Operative Variables

	SYL	DER	PRI	HIS	STE	COM	Total	*P*-value (with PRI)	*P*-value (without PRI)
Age	40.2 ± 16.3	43.7 ± 13.3	44.1 ± 16	42.2 ± 16.6	45 ± 17.8	45.1 ± 18.6	40.2 ± 16.1	.43	.80
BMI	31.6 ± 5.2	32.7 ± 5.5	36.6 ± 6.8	31.3 ± 5.7	30.8 ± 4.7	31.6 ± 5.6	32.0 ± 5.6	.00	.46
ASA	2 ± 0.5	2.1 ± 0.5	2.4 ± 0.5	2 ± 0.5	1.7 ± 0.6	2.2 ± 0.5	2 ± 0.5	.00	.16
Hypertension	22 (25.3)	4 (23.5)	4 (33.3)	21 (22.8)	4 (30.8)	9 (31.0)	64 (25.6)	.92	.33
Diabetes mellitus	3 (3.4)	4 (23.5)	0 (0.0)	6 (6.5)	2 (15.4)	5 (17.2)	20 (8.0)	.02	.40
Tobacco (current)	7 (8.0)	1 (5.9)	2 (16.7)	4 (4.3)	1 (7.7)	2 (6.9)	17 (6.8)	.71	.72
Tobacco (former)	5 (5.7)	2 (11.8)	1 (8.3)	12 (13.0)	2 (15.4)	6 (20.7)	28 (11.2)	.32	.18
Immunocompromised	4 (4.6)	1 (5.9)	0 (0.0)	5 (5.4)	0 (0.0)	0 (0.0)	10 (4.0)	.70	.12
Anticoagulation	3 (3.4)	1 (5.9)	0 (0.0)	1 (1.1)	0 (0.0)	2 (6.9)	7 (2.8)	.52	.61
Previous radiation therapy	5 (5.7)	0 (0.0)	0 (0.0)	10 (10.9)	0 (0.0)	4 (13.8)	19 (7.6)	.23	.26
Previous skin glue	19 (21.8)	2 (11.8)	4 (33.3)	25 (27.2)	4 (30.8)	10 (34.5)	64 (25.6)	.52	.26
Operative time	164.5 ± 56.6	157.2 ± 91.1	183 ± 38	167.3 ± 73.1	196 ± 61.8	223 ± 109.4	174.3 ± 74.6	.00001*	.06
Resection weight	622.4 ± 400	546.3 ± 372	1189.8 ± 393	535.8 ± 360	667.7 ± 630	583.9 ± 396	615.9 ± 421.8	.00001*	.59
Drain usage	18	3	7	31	2	10	71	.11	.005*
Total	87	17	12	92	13	29	250	—	—

ASA, American Society of Anesthesiologist class; COM, combination; DER, Dermabond; HIS, Histoacryl; PRI, Prineo; STE, Steri-Strip; SYL, Sylke.

^*^ *P* < .05; statistically significant.

Across all breasts, major complications occurred in 7.1% and minor complications in 34.0%. The most common events were wound dehiscence (17.8%), skin reaction (9.1%), infection (5.2%), and hematoma (5.7%). The most popular dressing used was HIS (37.8%), followed by SYL (34.6%), COM (11.2%), DER (5.9%), STE (5.5%), and PRI (5.0%). For the combination dressings, the most popular mix was SYL/HIS (38.8%), followed by SYL/STE (36.7%), SYL/DER (12.2%), HIS/STE (8.2%), and HIS/DER (4.1%).

### Bivariate Analysis

Initial analysis comparing all 6 dressing types revealed significant differences in BMI (*P* < .01), diabetes prevalence (*P* < .01), resection weight (*P* < .001), and operative time (*P* < .001) across groups, driven by the PRI cohort. PRI was also associated with higher rates of reoperation (*P* < .001), minor complications (*P* < .001), skin reaction (*P* < .001), hematoma (*P* < .01), dehiscence (*P* < .001), and scar complications (*P* < .001) compared with other dressing types.

As a sensitivity analysis to evaluate the impact of baseline disparities, a secondary analysis excluding PRI was performed. Among the remaining 5 dressings, no significant differences were found regarding patient demographics or operative details except for a higher drain usage in the HIS cohort (*P* < .01). Postoperative complication differences persisted for skin reaction (*P* < .001), wound dehiscence (*P* < .001), and scar complications (*P* < .001), and subsequent post hoc analysis revealed that SYL and COM dressings were associated with lower rates of skin reaction, dehiscence, and scar complications compared with other dressings (*P* < .05). The bivariate analysis results are represented in Table 2.

**Table 2.** Postoperative Complication Rates Per Breast (439)

	SYL (152)	DER (26)	PRI (22)	HIS (166)	STE (24)	COM (49)	Total (439)	*P*-value (with PRI)	*P*-value (without PRI)
Reoperation	5 (3.3)	1 (3.8)	8 (36.4)	13 (7.8)	1 (4.2)	3 (6.1)	31 (7.1)	.001*	.38
Any minor complication	48 (31.6)	5 (19.2)	15 (68.2)	64 (38.6)	8 (33.3)	9 (18.4)	149 (33.9)	.0007*	.09
Skin reaction	5 (3.3)	1 (3.8)	8 (36.4)	22 (13.3)	3 (12.5)	1 (2.0)	40 (9.1)	.001*	.001*
Hematoma/seroma	8 (5.3)	2 (7.7)	0 (0.0)	13 (7.8)	1 (4.2)	1 (2.0)	25 (5.7)	.51	.24
Wound dehiscence	19 (12.5)	2 (7.7)	10 (45.5)	36 (21.7)	4 (16.7)	7 (14.3)	78 (17.8)	.0025*	.001*
Skin infection	7 (4.6)	2 (7.7)	4 (18.2)	9 (5.4)	0 (0.0)	1 (2.0)	23 (5.2)	.07*	.42
Antibiotic prescription	7 (4.6)	2 (7.7)	4 (18.2)	9 (5.4)	0 (0.0)	2 (4.1)	24 (5.5)	.11	.39
Necrosis	1 (0.7)	0 (0.0)	1 (4.5)	2 (1.2)	0 (0.0)	0 (0.0)	4 (0.9)	.49	.38
Poor scarring	3 (2.0)	0 (0.0)	11 (50.0)	6 (3.6)	1 (4.2)	0 (0.0)	21 (4.8)	.0001*	.001*

COM, combination; DER, Dermabond; HIS, Histoacryl; PRI, Prineo; STE, Steri-Strip; SYL, Sylke.

^*^ *P* < .05; statistically significant.

### Multivariate Analysis

Multivariate regression was performed to evaluate independent predictors of postoperative complications while controlling for BMI, American Society of Anesthesiologist class, diabetes mellitus, resection weight, operative time, and drain use. Variables were selected using an enter-all method that included all factors with a univariate *P*-value of ≤.20.

In the 6-group model, PRI use was independently associated with higher odds of reoperation (odds ratio [OR] 6.43, 95% CI, 1.51-28.57, *P* = .01) and scar complications (OR 34.85, 95% CI, 6.10-252.98, *P* < .001). In contrast, SYL (OR 0.21, 95% CI, 0.07-0.54, *P* = .003) and COM (OR 0.08, 95% CI, 0.00-0.45, *P* = .02) dressings were associated with significantly lower odds of skin reactions, and SYL also reduced the odds of dehiscence (OR 0.48, 95% CI, 0.25-0.88, *P* = .02).

After excluding PRI, SYL continued to show decreased odds of skin reaction (OR 0.20, 95% CI, 0.06-0.53, *P* = .003) and dehiscence (OR 0.48, 95% CI, 0.25-0.88, *P* = .02), whereas COM also reduced odds of skin reaction (OR 0.08, 95% CI, 0.00-0.45, *P* = .02). Increasing operative time independently predicted greater risk of reoperation (OR 1.01 per minute, 95% CI, 1.00-1.01, *P* = .005). All models demonstrated acceptable fit (McFadden's *R*^2^ = 0.05-0.17; Hosmer–Lemeshow *P* > .05). The multivariate analysis results are displayed in Tables 3 and 4.

**Table 3.** Skin Reaction Risk Factors^a^

	aOR (95% CI)	*P*-value
BMI	1.08 (0.99-1.19)	.082
ASA		
1	(Ref.)	(Ref.)
2	0.57 (0.18-1.96)	.338
3	0.93 (0.19-4.64)	.924
DM	2.24 (0.56-7.49)	.212
Resection weight	1.00 (0.99-1.00)	.524
Operative time	1.00 (0.99-1.01)	.358
Drain use	0.46 (0.16-1.19)	.132
Dressing		
HIS	(Ref.)	(Ref.)
SYL	0.20 (0.06-0.53)	.003*
DER	0.22 (0.01-1.24)	.159
STE	0.82 (0.16-3.18)	.789
COM	0.08 (0.00-0.45)	.020*

aOR, adjusted odds ratio; ASA, American Society of Anesthesiologist class; COM, combination; DER, Dermabond; HIS, Histoacryl; PRI, Prineo; STE, Steri-Strip; SYL, Sylke.

^*^ *P* < .05; statistically significant.

^a^PRI omitted.

**Table 4.** Wound Dehiscence Risk Factors^a^

	aOR (95% CI)	*P*-value
BMI	0.99 (0.94-1.07)	.991
ASA		
1	(Ref.)	(Ref.)
2	1.28 (0.55-3.42)	.586
3	2.03 (0.61-7.03)	.251
DM	0.98 (0.31-2.65)	.978
Resection weight	1.00 (0.99-1.00)	.077
Operative time	1.00 (0.99-1.01)	.694
Drain use	0.72 (0.37-1.33)	.305
Dressing		
HIS	(Ref.)	(Ref.)
SYL	0.48 (0.25-0.88)	.020*
DER	0.14 (0.01-0.73)	.062
STE	0.54 (0.11-1.91)	.378
COM	0.48 (0.17-1.17)	.127

aOR, adjusted odds ratio; ASA, American Society of Anesthesiologist class; COM, combination; DER, Dermabond; HIS, Histoacryl; PRI, Prineo; STE, Steri-Strip; SYL, Sylke.

^a^PRI omitted.

^*^ *P* < .05; statistically significant.

## DISCUSSION

Wound-dressing selection is an essential component of postoperative care in reduction mammaplasty, influencing both wound healing and patient comfort. Although multiple closure and dressing materials are available, comparative data specific to reduction mammaplasty remain limited. In this study, we compared the rates of reoperation and postoperative complications between 6 commonly used wound dressings. In 250 patients, totaling 439 breasts, we observed notable differences in reoperation rates and minor complications, particularly skin reactions, wound dehiscence, and poor scarring.

### Dermal Complications

In our cohort, patients treated with SYL dressings experienced significantly lower rates of skin reactions and wound dehiscence. These findings support previous work demonstrating fewer medical adhesive-related skin injuries (MARSIs) with SYL compared with synthetic dressings, such as PRI and STE. In 2023, Rouhani et al evaluated the rates of MARSI with the use of SYL vs PRI.^17^ In addition to fewer MARSIs, they found significantly lower rates of patient-reported discomfort, skin rash, and antibiotic use in the SYL cohort. Similarly, a study comparing SYL to STE following reduction mammaplasty, abdominoplasty, and brachioplasty reported lower rates of erythema, triple-point dehiscence, and improved dressing adherence in the SYL cohort.^18^ These findings extend beyond plastic surgery. A recent orthopedic study demonstrated reduced incidence of allergic contact dermatitis in patients undergoing total joint arthroplasty when SYL dressings were used instead of cyanoacrylate mesh.^20^

### Biomechanical and Chemical Considerations

The biomechanical and chemical properties of synthetic dressings may contribute to their increased rates of wound complications. Both HIS (*n*-butyl-2-cyanoacrylate) and DER (2-octyl cyanoacrylate) belong to the cyanoacrylate class of topical skin adhesives. *N*-butyl formulations form a more rigid adhesive and have demonstrated inferior adhesive strength in multiple studies, which may contribute to higher rates of wound dehiscence.^21,22^ PRI additionally incorporates a polyester mesh containing benzalkonium chloride, a catalyst used to accelerate curing. Benzalkonium chloride, a known irritant, may partially explain the higher rates of skin reaction observed with PRI in our cohort.^23^

Upon application, cyanoacrylates rapidly polymerize with moisture to form a solid film that subsequently degrades into cyanoacetate and formaldehyde.^24^ A recent study in Contact Dermatitis found that 2-octyl cyanoacrylate adhesives release formaldehyde at dermal and airborne levels exceeding United States Environmental Protection Agency and National Institute for Occupational Safety and Health thresholds by up to 8.4- and 2.41-fold, respectively, supporting a plausible mechanism for Type IV hypersensitivity and postoperative skin reactions.^25^ A recent systematic review and meta-analysis published in the *Journal of the American College of Surgeons* included 74 studies and 26,330 exposed patients and reported a pooled allergic contact dermatitis incidence of 4% with cyanoacrylate adhesives, with higher rates in plastic surgery (8%) than orthopedic surgery (4%). Reexposure was among the strongest risk factors, with reaction rates rising from 1% to 22% in staged bilateral arthroplasty, whereas previous sensitization from consumer products, such as artificial nails, eyelash adhesives, and super glues, was also an important contributor to subsequent reactions. These findings provide broader clinical context for the higher skin reaction rates observed with cyanoacrylate-based dressings in our cohort.^26^

In contrast, silk fibroin is a nonimmunogenic protein with favorable mechanical strength, biocompatibility, and biodegradability.^27^ Its lattice-like structure allows controlled expansion with tissue movement, reducing tension and preventing microtears at the incision site.

We believe these biomechanical and biochemical characteristics explain the improved performance of SYL dressings in our study. The lower rates of skin reaction observed in the COM cohort can similarly be attributed to the high proportion of silk-based material used, as 87.8% of combinations incorporated SYL. Conversely, we hypothesize that liquid adhesives, whereas forming a protective microbial barrier, may hinder the natural apposition of wound edges when excess material interposes between them.

### Limitations and Future Directions

This study is limited by its retrospective, single-institution design. Dressing selection was based on surgeon preference, introducing potential selection bias and unmeasured confounders. Although surgeon preference was the dominant driver of dressing selection, additional clinical factors such as wound tension, skin quality, extent of resection, pedicle type and incision pattern may have also influenced these decisions on a case-by-case basis. Because these variables were not captured, they may represent additional unmeasured confounders. Additionally, the PRI cohort had nearly double the resection weight as well as the highest BMI average. These high-risk patient characteristics introduce multiple confounders, and the PRI results should be interpreted with caution. Scar outcomes were assessed retrospectively from clinical follow-up documentation, and the ∼3-month follow-up period may not fully reflect longer-term scar maturation or final cosmetic appearance. The combination dressing group warrants particular interpretive caution because it encompasses a heterogenous range of dressings, and it is difficult to attribute outcomes to any single variable. Although this group may reflect real-world practice patterns, the variability inherent to its composition limits the conclusions that can be drawn. The secondary analysis excluding the PRI cohort was performed as a sensitivity analysis rather than a definitive correction for baseline differences. Additionally, uneven cohort sizes may limit the power of certain comparisons, particularly for smaller groups such as PRI. Small subgroup sizes also resulted in wide CIs for certain estimates, reflecting potential imprecision and limiting the stability of effect size estimates. Although generalized estimating equations or propensity score-based methods may further reduce bias, their application in this study was limited by small subgroup sizes and multiple treatment arms. Prospective, randomized, multi-institutional studies are needed to validate these findings and clarify optimal dressing selection.

## CONCLUSIONS

Dressing selection may affect wound outcomes after reduction mammaplasty. SYL tape was associated with lower rates of skin reaction and dehiscence across both analyses, although not all potential confounding variables could be ruled out as contributing to the study findings. PRI's association with reoperation and poor scarring is notable but difficult to disentangle from patient selection bias. The combination group showed lower skin reaction rates, although its heterogeneity makes interpretation challenging. Prospective data are needed to confirm these findings.
